# Can bipolar energy serve as an alternative to monopolar energy in the management of large bladder tumours >3 cm? A prospective randomised study

**DOI:** 10.1080/2090598X.2019.1590517

**Published:** 2019-04-23

**Authors:** Mahmoud A. Mahmoud, Ahmed Tawfick, Diaa Eldin Mostafa, Hossam Elawady, Mohamed Abuelnaga, Karim Omar, Hisham Elshawaf, Mohamed Hasan

**Affiliations:** Department of Urology, Ain Shams University Hospital, Cairo, Egypt

**Keywords:** Transurethral resection of bladder tumour (TURBT), bipolar TURBT, monopolar TURBT

## Abstract

**Objectives**: To assess the safety and the effectiveness of bipolar energy in the transurethral resection of primary large bladder tumours (TURBT) and compare it to conventional monopolar energy.

**Patients and methods**: From November 2015 to June 2017, 80 patients underwent endoscopic resection primarily for large bladder cancer tumours of >3 cm. They were randomly assigned into two groups: 40 patients underwent a TURBT with conventional monopolar current (M-TURBT) and 40 were treated with bipolar current (B-TURBT).

**Results**: There were no statistically significant differences between the two groups for the patients’ demographic and tumour characteristics. There was a significant difference between M-TURBT and B-TURBT for resection time, obturator reflex, hospital stay, and catheterisation time, which were all higher in the M-TURBT group; the mean (SD) resection time was 26.45 (5.73) vs 22.85 (7.52) min (*P* = 0.048), the obturator reflex was 25% vs 5% (*P* = 0.025), the median hospital stay and catheterisation times were 2 vs 1 day (*P* = 0.012 and *P* = 0.023, respectively). No statistically significant difference was found between the groups for bladder perforation, TUR syndrome, drop in haemoglobin level, and blood transfusion rate. However, there was statically significant difference in the postoperative haematuria rate, which was higher in the M-TURBT group, at 24 patients vs eight in the B-TURBT group (60% vs 20%; *P* = 0.01). After 1-year follow-up, there was no significant difference in the recurrence rate between the groups.

**Conclusion**: B-TURBT is a safe and effective alternative procedure to M-TURBT for the management of primary large bladder tumours of >3 cm.

**Abbreviations:** CONSORT: consolidated standards of reporting Trials; Hb: haemoglobin; NMIBC: non-muscle-invasive bladder cancer; TUR: transurethral resection; (B-)(M-)TURBT: (bipolar) (monopolar) transurethral resection of bladder tumour

## Introduction

Bladder cancer is the fourth most common cancer in men and the eighth most common in women worldwide. It is the second most common malignancy affecting the urinary system after prostate cancer [1].

Urothelial carcinomas (UC) comprise up to 90% of all primary bladder tumours. Around 70–75% of UC present as non-muscle-invasive bladder cancer (NMIBC), whilst 25–30% are muscle invasive (≥pT2) at presentation [2].

Transurethral resection (TUR) is considered the ‘gold standard’ surgical technique for management of bladder tumours [3]. TUR of bladder tumour (TURBT) using monopolar current (M-TURBT) as the source of energy, is the standard of care [4]. However, one of the risks of using hypotonic fluid during M-TURBT is TUR syndrome, although this is more relevant during TUR of the prostate [5]. Also, electric current can easily stimulate the obturator nerve and cause adductor reflex during resection of laterally situated bladder tumours. The obturator nerve reflex may cause intraoperative complications in terms of bladder wall perforation, excessive bleeding, and thus cause incomplete resection and a longer hospital stay [6].

One of the more recent advances in the field of urology has been the incorporation of bipolar technology for TUR [7]. This technology allows resection to be performed in the presence of normal saline, which helps avoid the occurrence of TUR syndrome [3]. Moreover, the theoretically closed circuitry of the bipolar system is claimed to reduce obturator nerve stimulation and bladder perforation [8].

That said, in the present study, we aimed to assess the safety and effectiveness of bipolar energy in the TUR of primary large bladder tumours (B-TURBT) and compare it to conventional M-TURBT.

## Patients and methods

The sample size was calculated using the PASS program version 11, setting the type-1 error (α) at 0.05 and the power (1-β) at 0.8. A previous study reported that in the M-TURBT group obturator reflex was seen in 26.5%, whilst in the B-TURBT group, obturator reflex was seen in 4.8%. Calculation according to these values produced a sample size of 39 in each group [9].

Between November 2015 and June 2017, 235 patients presented with bladder tumours to the Department of Urology at Ain Shams University Hospital. Of those, 80 patients were included in the study based on the following inclusion and exclusion criteria:

Inclusion criteria:

- Newly diagnosed primary bladder tumours, with tumour size >3 cm.

Exclusion criteria:

- Unfitness for spinal anaesthesia.
- Patients with recurrent bladder tumour.
- Patients with other urological malignancies.
- Patients requiring anticoagulation.
- Patients with pacemakers.
- Patients with back pressure change.
- Patients with urethral stricture.
- Active UTIs.
- Patients with uncontrolled bleeding diathesis.

The patients were fully informed about the procedures and chances of success and complications. Patients were randomised according to a simple 1:1 randomisation and alternated between the two groups. Group A patients underwent M-TURBT and Group B patients B-TURBT.

**Figure 1.**
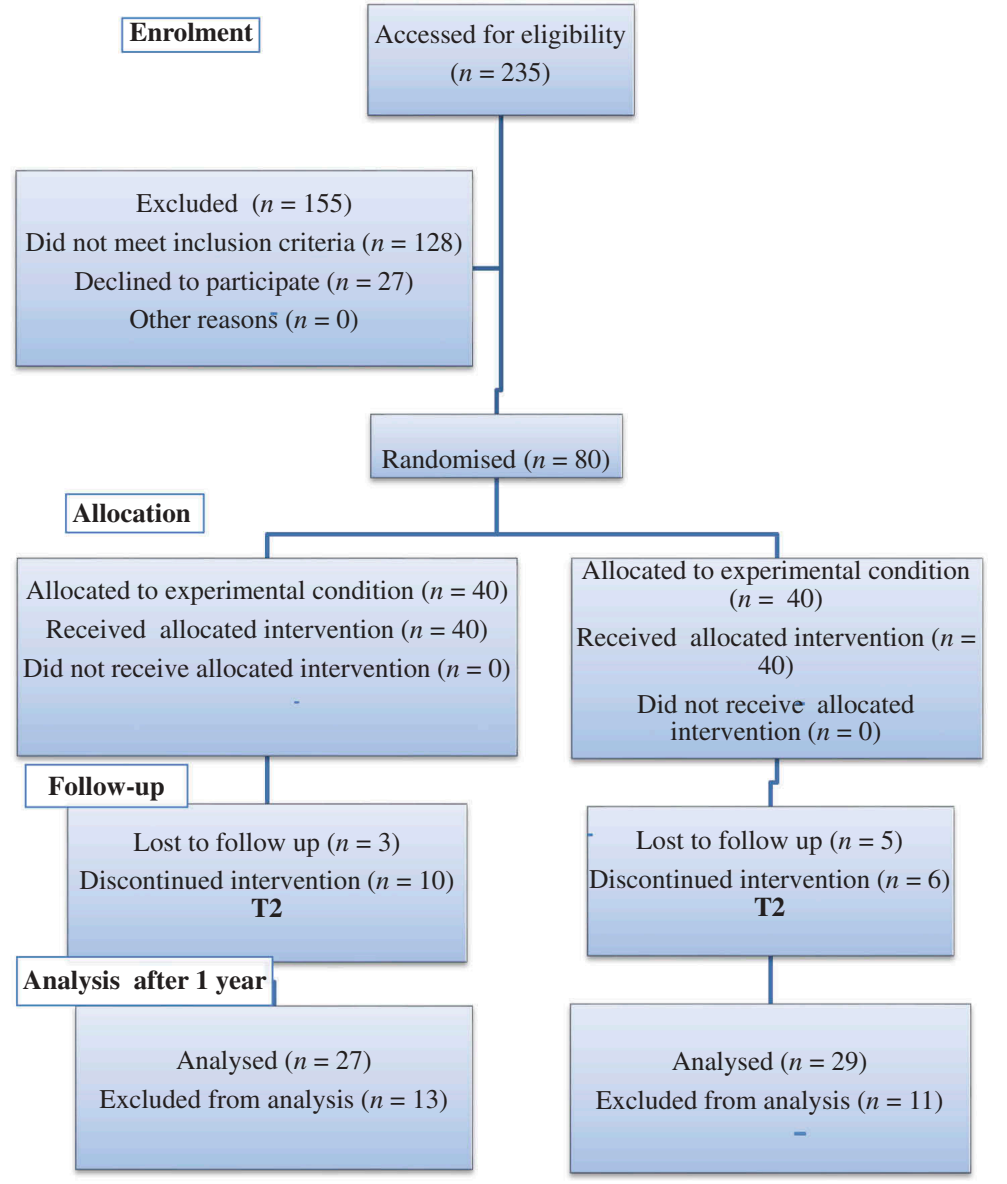
Consort flow chart.

All patients were assessed preoperatively with a detailed medical history, physical examination, urine analysis, urine culture, renal ultrasonography and a CT of the urinary tract after contrast injection, as well as a routine preoperative evaluation. The study design and workflow are summarised in the Consolidated Standards of Reporting Trials (CONSORT) flow chart (Figure 1). The study design was an intention-to-treat analysis.

We assessed the effectiveness through resection time, catheterisation time and hospital stay, as well as safety by obturator nerve reflex, bladder perforation, haemoglobin (Hb) decrease, and transfusion rate. The primary endpoint was to investigate the safety of B-TURBT vs M-TURBT. The secondary endpoint was to evaluate the recurrence rate of both approaches.

All procedures were carried out under spinal anaesthesia, in a lithotomy position. The conventional M-TURBT was carried out using a Storz 24-F resectoscope with continuous flow and a U-shaped cutting loop; 1.5% glycine was used as the irrigant. Once the device was connected, the generator was programmed to 70 W for both cutting and coagulation. Whilst in B-TURBT, the procedure was performed with the Olympus transurethral resection in saline (TURis) system with a continuous-flow 24-F resectoscope and a U-shaped cutting loop; 0.9% normal saline was used as the irrigant. Once the device was connected, the generator was programmed to 70 W for cutting and 80 W for coagulation.

After the complete removal of visible bladder tumour, a 20-F three-way catheter was inserted. Continuous bladder irrigation was maintained until the efflux was clear, and the catheter was removed when the urine was clear. All patients underwent an early instillation of mitomycin 40 mg within 24 h of their TURBT.

All resected specimens were submitted to the Department of Pathology, fixed in formalin, sectioned, and stained with haematoxylin and eosin. Pathologists evaluated tissues for tumour stage, WHO grade, presence of lamina propria, the presence of deep muscle in the sample, and muscle invasion.

For all patients, the following data were recorded: patient age, gender, tumour size, tumour location, tumour number, resection time (from the period of initiation of resection to the removal of resectoscope sheath), all perioperative complications, changes in Hb, catheterisation time, duration of hospital stay, pathological stage, WHO grade, and recurrence rate. Follow-up cystoscopy for tumour recurrence was done every 3 months up to 1 year and it was completed only in 27 patients in Group A and 29 patients in Group B.

### Statistical analysis

The collected data were revised, coded and entered in to the IBM Statistical Package for the Social Sciences (SPSS®), version 20 (SPSS Inc., IBM Corp., Armonk, NY, USA). Quantitative data are presented as means, standard deviations (SDs) and ranges, whilst qualitative data are presented as numbers and percentages. Comparisons between the two groups were done using the Wilcoxon Mann–Whitney test for non-parametric continuous variables and Student’s *t*-test for parametric quantitative variables, whilst qualitative data were compared using the chi-squared test or, alternatively, the Fisher’s exact test when the expected count in any cell was found to be <5. The CI was set to 95% and the accepted margin of error was set to 5%. The *P* value was considered statistically significant at *P* ≤ 0.05.

### Ethical considerations

The present study was approved by Faculty of Medicine’s Ethics Committee. Written informed consent was obtained from all patients.

## Results

There were no statistically significant differences between the two groups for the patients’ demographic and tumour characteristics (Table 1).

**Table 1.** Patients’ demographic and tumour characteristics.

Variable	M-TURBT, *n* = 40	B-TURBT, *n* = 40	*P*
Age, years, mean (SD; range)	58.85 (10.07; 40.0–77.0)	59.35 (9.53; 35.0–71.0)	0.873
Gender, *n* (%) Female Male	36 (90) 4 (10)	30 (75) 10(25)	0.407
Tumour size, cm, mean (SD; range)	4.06 (0.76; 3.0–5.0)	4.09 (0.84; 3.0–6.0)	0.923
Tumour multiplicity, *n*	5	7	1.000
Tumour location, *n* Dome Trigone LL wall RL wall RPL wall LPL wall Post wall	6 4 6 4 16 10 4	4 6 4 6 12 16 2	1.000 1.000 1.000 1.000 0.507 0.311 1.000
Pathology and grade, *n* (%) Low TCC High TCC	14 (35) 26 (65)	18 (45) 22 (55)	0.519
Stage, *n* (%) Ta T1 T2	8 (20) 22 (55) 10 (25)	12 (30) 22 (55) 6 (15)	0.641

LL, left lateral; RL, right lateral; RPL, right posterolateral; LPL, left posterolateral. There were no statistically significant differences between the groups.

Intraoperatively there was a significant difference between M-TURBT and B-TURBT in the mean (SD) resection time, which was higher in the M-TURBT group, at 26.45 (5.73) vs 22.85 (7.52) min (*P* = 0.048). Also the obturator reflex was higher in the M-TURBT group, at 25% vs 5% (*P* = 0.025). There were no statistically significant differences between the groups regarding bladder perforation and TUR syndrome. No patient in either group required a blood transfusion (Table 2). Extraperitoneal bladder perforation occurred in two patients in the M-TURBT group. They were treated conservatively with prolonged catheter drainage. TUR syndrome developed in one patient in the M-TURBT group. The patients were all aged 66 years, and resection took 36 min. The patients were managed with 80 mg i.v. furosemide.

**Table 2.** Intra- and postoperative data.

Variable	M-TURBT, *n* = 40	B-TURBT, *n* = 40	*P*
**Intraoperative data**			
Resection time, min, mean (SD; range)	26.45 (5.73; 20.0–36.0)	22.85 (7.52; 15.0–38.0)	0.048
Obturator reflex, *n* (%)	10 (25)	2 (5)	0.025
Bladder perforation, *n* (%)	2 (5)	0	0.487
TUR syndrome, *n* (%)	1 (2.5)	0	1.000
**Postoperative data**			
Hospital stay, days, median	2	1	0.023
Catheterisation time, days, median	2	1	0.012
Haematuria, *n* (%)	24 (60)	8 (20)	0.010
Drop in Hb, g/dL, mean (SD)	1.28 (0.67)	1.32 (0.50)	0.830

There were statistically significant differences between the groups for the resection time, obturator reflex, hospital stay, catheterisation time, and postoperative haematuria.

Postoperatively there was a statistically significant difference in the haematuria rate, which was higher in the M-TURBT group, occurring in 24 patients vs eight in B-TURBT group (60% vs 20%, *P* = 0.01), but there was no need for blood transfusion in either group. There was no significant difference between M-TURBT and B-TURBT for drop in Hb level; at a mean (SD) of 1.28 (0.67) g/dL in the M-TURBT group and 1.32 (0.50) g/dL in the B-TURBT group (*P* = 0.830). The catheterisation time and hospital stay were significant lesser in the B-TURBT group (*P* = 0.012 and *P* = 0.023, respectively) (Table 2).

Regarding the recurrence rate, after 1-year follow-up with cystoscopy every 3 months, there was no significant difference between the groups; seven of 27 patients in Group A and nine of 29 patients in Group B (*P* = 0.77).

## Discussion

Bladder cancer is the most common urinary tract malignancy and 75–80% of cases are NMIBCs at diagnosis [10].

TURBT is the ‘gold standard’ procedure for the diagnosis and treatment of NMIBC. The aim of initial resection is to remove all visible tumours including part of the underlying muscle of the bladder [7].

Previously, TURBT was performed conventionally with monopolar loop electrocautery employing non-saline irrigation fluid with its inadvertent potential hazards of hypotonic fluid absorption and ensuing electrolyte imbalance [11]. Also, the obturator reflex can occur when the obturator nerve is directly stimulated by the electrical current transmitted by the resectoscope, especially when the tumour is localised at the lateral wall of the bladder [12]. Recently, bipolar energy has been used for TURBT. There have been several reports of favourable results for B-TURBT, including decreased bladder perforation due to less tissue depth, better haemostasis, and shorter hospital stay [13].

The main advantage of bipolar electrocautery is less tissue charring and blackening. Excellent visualisation of anatomy with bipolar resection allows controlled resection and avoids damaging of adjacent structures. Another advantage of bipolar energy becomes apparent when treating high-risk patients with bladder tumours, such as those with implanted pacemakers and pregnant women [14].

Regarding our first endpoint on the safety of the procedure in our present study, the perioperative complication rate in the M-TURBT group was statistically insignificant compared to the B-TURBT group. Comparable results were obtained by Liem et al. [5] and Del Rosso et al. [7], who reported that there was no statistically significant difference in intra- and postoperative complications between monopolar and bipolar current. Conversely, Geavlete et al. [15] in their randomised study reported more frequent perioperative complications in patients treated with M-TURBT. The same results were obtained by Xishuang et al. [16], reporting fewer intra- and postoperative complications for the B-TURBT.

TUR syndrome after M-TURBT is rarely reported, with an incidence of ~2% [17]. The rarity of this complication led to the absence of a significant difference between both techniques in clinical TUR syndrome between monopolar and bipolar tumour resection [16,18]. These data were comparable to our present study, where no patient developed TUR syndrome in the B-TURBT group and only one patient (2.5%) in the M-TURBT group developed TUR syndrome, which was overall statistically insignificant. Also, these data corresponds to the Del Rosso et al. [7] study, which reported that no patient in either group developed TUR syndrome.

Bladder injury in TURBT is rare and it has a high risk of extravesical tumour seeding [19]. The incidence and the differences between both techniques in inducing bladder perforation is an area of heated debate and confusion. In our present study, no patients developed bladder peroration in the B-TURBT group but it affected two patients (5%) in the M-TURBT group. However, there was no statistically difference between the groups (*P* = 0.487). Comparable results were obtained by Del Rosso et al. [7], whereby bladder perforation was reported in two cases in the monopolar arm and in none in the bipolar arm, with no statistically difference between the groups. Hashad et al. [20] also reported bladder perforation in four patients in their monopolar arm and in one patient in the bipolar arm, with no statistical difference between the groups (*P* = 0.369). Mashni et al. [21] reported that no patients developed bladder perforation, with no statistically difference between the groups. Also Liem et al. [5] reported no statistically difference between the groups. Venkatramani et al. [18] reported that bladder injury was higher in their bipolar group but was not statistically significant. They also concluded that B-TURBT was not superior to M-TURBT with respect to bladder perforation.

Sugihara et al. [22] reported that the incidence of bladder injury was significantly higher in M-TURBT (0.3% vs 0.6%). This study clearly establishes the superiority of B-TURBT. Also, Mansour et al. [9], in a controlled randomised trial, reported a 13.2% perforation rate with monopolar resection that was significantly higher than the 2.4% for bipolar resection (*P* = 0.02). Conversely, Ozer et al. [23], reported that bladder injury was statistically significantly higher in the bipolar group, with bladder perforation seen in 10 (23%) patients vs four (8%) (*P* = 0.4). Furthermore, in another study, it was reported that a significant rate of obturator jerks and subsequent perforation in their first 10 patients when the power setting of the bipolar machine was adjusted to 160 and 80 W for cutting and coagulation, respectively. They showed that such complications were eliminated by using lower power settings of 50 and 40 W, respectively [24].

Obturator reflex has stimulated much debate and confusion because some reports described the occurrence of obturator reflex in nearly half of the patients [18], whilst others reported an incidence of ~1% [7]. In our present study, obturator reflex was higher in the M-TURBT group, affecting 10 patients (25%) vs two (5%) in the B-TURBT group, which was statistically significant (*P* = 0.025). Mashni et al. [21], Hashad et al. [20], Del Rosso et al. [7] and Liem et al. [5] reported equal incidence between both techniques, at 4 vs 4, 15 vs 12, 1 vs 1, and 22 vs 25, respectively. The Xishuang et al. [16] report favoured B-TURBT in abolishing the obturator reflex in a statistically significant way compared with M-TURBT. Also, Mansour et al. [9] reported a significant decrease in the incidence of nerve stimulation from 26.5% with M-TURBT vs 4.8% with B-TURBT (*P* = 0.01). Conversely, Ozer et al. [23] reported that the obturator reflux was statistically significantly higher in B-TURBT group; with the obturator reflex observed in 15 (34%) patients vs four (8%) (*P* = 0.001). Also, in the Venkatramani et al. [18] report, the incidence of obturator jerk was greater in the B-TURBT arm (60% vs 49.2%, *P* = 0.27). Both concluded that B-TURBT was not superior to M-TURBT with respect to obturator jerk and bladder perforation.

Multiple studies have reported that the resection time was equal in both arms regardless of the type of energy used [5,7,9,16,18,21,22]. In our present study, there was a significant difference between M-TURBT and B-TURBT for resection time, which was greater in the M-TURBT group, at a mean (SD) of 26.45 (5.73) vs 22.85 (7.52) min. Utilising B-TURBT was expected to reduce resection time due to reduced adhesions of residual fragments to the loop of a resectoscope, which are easily removed by acting on the cutting function with the loop in the bladder lumen. In contrast, the monopolar device requires a manual and mechanical removal of the residual chips through extraction of the instrument from its sheath, which prolongs the procedure [5].

In our present study, we found a statically significant difference in the postoperative haematuria rate, which was higher in the M-TURBT group (60% vs 20%, *P* = 0.01); however, neither group required blood transfusion. Also, there was no significant difference between M-TURBT and B-TURBT for the drop in Hb level. Yang et al. [25] reported a significant drop in Hb level with M-TURBT vs B-TURBT, which was not reflected in the transfusion rate. Also, Hashad et al. [20] reported that the postoperative reduction in Hb concentration was significantly lower in the B-TURBT group vs the M-TURBT group (P < 0.001). There was a significant difference (in favour of B-TURBT) between the groups in the mean postoperative reduction in haematocrit but there was no significant difference between the groups in requirement for blood transfusion. Equally, others have reported that the bleeding events, transfusion rates, need for re-coagulation, and decrease in packed cell volume were comparable between both techniques [5,7,16,22].

Better control of intraoperative bleeding allows for a shorter postoperative period of catheterisation with a subsequently faster discharge [7]; also, the present results show shorter hospitalisation duration for B-TURBT. There is universal agreement that the use of bipolar resection decreases the hospital stay [7,16,20,22], as with bipolar current there is the more efficient property of bipolar resection in cutting and simultaneously controlling bleeding, when compared with the monopolar procedure [26]. On the other hand Liem et al. [5] found no statistically significant difference between the groups.

Del Rosso et al. [7] in a randomised clinical trial showed a significant reduction in the mean (range) hospitalisation time with B-TURBT at 2.2 (2–7) days vs 3.5 (3–6) days in the M-TURBT group (*P* = 0.008). Hashad et al. [20] also reported a significant difference in the mean (SD) postoperative hospital stay in favour of B-TURBT at 31.20 (11.57) vs 42.24 (15.67) h for M-TURBT (*P* < 0.001) [20]. In our present study, the hospital time was also shorter in the B-TURBT group at 1 day vs the M-TURBT group at 2 days. Conversely, Ozer et al. [23] reported that the mean (SD) hospitalisation time was statistically significantly lower in the monopolar arm at 1.58 (1.03) vs 2.09 (1.17) days in the bipolar arm (*P* = 0.001).

Del Rosso et al. [7] also reported a significant reduction in the catheterisation time with B-TURBT, at 1.3 vs 2.3 days in the monopolar arm (*P* = 0.01). In our present study, the catheterisation time was also significantly lower in the B-TURBT group at 1 day vs the M-TURBT group at 2 days. Conversely, Ozer et al. [23] reported that the mean (SD) catheterisation time was lower in the monopolar arm at 4.34 (1.62) vs 4.62 (1.79) days in the bipolar arm, but this was not statistically significant (*P* = 0.17).

With regard the second endpoint, there are limited data on the recurrence rate following bipolar energy use. In our present study, there is no difference between the groups regarding the rate of recurrence of bladder tumour over the 1-year follow-up. Other studies have also reported that there was no impact on the recurrence rate no matter the type of energy utilised [5,7,16].

There are certain limitations in our present study: e.g. we did not analyse serum electrolyte levels, or assess the cautery artefacts in pathological specimens. The oncological outcomes were short-term, and finally, we were confined by the single-centre nature of the study.

## Conclusion

B-TURBT, compared with M-TURBT, appears to be a safe endoscopic treatment with advantages in resection time, obturator reflex, hospital stay, catheterisation time and postoperative haematuria for large bladder tumours >3 cm.
